# Identification of colistin resistance and its bactericidal activity against uropathogenic gram negative bacteria from Hayatabad Medical Complex Peshawar

**DOI:** 10.12669/pjms.38.4.5221

**Published:** 2022

**Authors:** Ambreen Arif, Ihsan Ullah, Obaid Ullah, Ronaq Zaman

**Affiliations:** 1 Ambreen Arif Research Officer, Health Research Institute, NIH (Islamabad). Khyber Medical College Peshawar, Khyber Medical University, Peshawar, Pakistan; 2 Dr. Ihsan Ullah, Associate Professor (Microbiology), Khyber Medical University, Peshawar, Pakistan.; 3 Obaid Ullah, Senior Research Officer, Health Research Institute, NIH (Islamabad). Khyber Medical College Peshawar, Pakistan; 4 Ronaq Zaman, Assistant Professor, Kabir Medical College, Peshawar, Pakistan

**Keywords:** Antibiotics, Colistin Resistance, Gram-Negative Bacteria, Minimum Inhibitory Concentration, Urinary Tract Infection

## Abstract

**Objectives:**

Identification of colistin resistance and its bactericidal activity against gram-negative bacteria isolated from urinary tract infection (UTI) patients.

**Methods:**

This 6-month cross sectional study was conducted in Hayatabad Medical Complex Peshawar from January 2019-June2019.. A total of 2000 urine samples were collected and transported to the Health Research Institute, NIH, Research Centre, Khyber Medical College Peshawar. Samples were streaked on different media and incubated at 37C° for 24hrs. Gram negative bacteria were identified through gram staining and Analytical Profile Index (API) 10s. Gram negative bacteria were subjected under antibiotic sensitivity profile through Kirby-Bauer disc diffusion method. Colistin resistance was found through broth microdilution method. Minimum bactericidal activity was performed to find out the lowest concentration of colistin required to kill gram-negative bacteria.

**Results:**

A total of 241(12.05%) uropathogenic gram negative bacteria were isolated and identified from 2000 urine samples while excluding intrinsically resistant bacteria. After broth microdilution, colistin resistance was found in 48(19.9%) *Escherichia coli*, 4(1.6%) *Klebsiella pneumoniae* and 3(1.3%) *Pseudomonas aeruginosa* respectively. Colistin resistant *Escherichia coli were resistant to* 77% Cephalosporins*, 81% to* Fluoroquinolones and 70% to Penicillin combinations. Colistin resistant *Klebsiella pneumoniae were 100%* resistant to Cephalosporins, Penicillin combinations and Fluoroquinolones while 75% were resistant to Carbapenems and Monobactams. *Pseudomonas aeruginosa* isolates were sensitive to all used antibiotics.

**Conclusion:**

*E.coli* was the mainly responsible uropathogen causing UTIs. Colistin resistance was found in 22.8% gram negative uropathogens. *Klebsiella pneumoniae* isolates exhibited highest resistance to antibiotics.

## INTRODUCTION

The prevalence of urinary tract infection (UTI) caused by multi-drug resistant gram negative bacteria is a key issue around the world, as these resistant bacteria developed the resistance to fluoroquinolone, cephalosporins and carbapenems.^1^ Different microorganisms can cause UTI but 95% of cases are caused by bacteria.^2^ Among uropathogenic bacteria, gram negative specially Enterobacteriaceae is mainly responsible for UTI. Among Gram Negative Bacteria, *Escherichia coli* (70-80%) is the major reason for UTI.^3^

Resistance to fluoroquinolones, β-lactams, aminoglycosides and carbapenems and the lack of development of new antibacterial drugs lead the medical community to reconsider the colistin for the treatment of Multidrug resistant gram negative bacteria worldwide.^4^,^5^

Colistin is an old antibiotic, which also known as polymyxin E antibiotic which was approved by the FDA in 1959.^6^ It is widely used for the treatment of animals and now increasingly given worldwide to human for the treatment of multi drug resistant bacteria e.g. gram-negatives.^7^

Colistin have narrow spectrum activity, as it is active against gram negative bacteria mainly Enterobacteriaceae and some non-fermentative Gram-negative bacteria e.g., *P. aeruginosa* and A.baumannii.^8^

Unfortunately development of resistance to colistin was also observed in gram negative bacteria. Even though the prevalence of colistin resistance is low around the world, and found in different multi drug resistant gram negative bacteria such as *E.coli, K. pneumoniae, P. aeruginosa and A. baumannii*.^9^ Several mechanisms are involved in resistance of colistin e.g. intrinsic, adaptive and mutational and horizontally acquired resistance via plasmid.^10^

The problem in phenotypic detection of colistin resistance is due to interactions of this antibiotic with materials e.g. cations, make it hindered for global surveillance of antimicrobial resistance of colistin.^11^ Colistin diffusion in agar media is uneven and irregular and interaction in media make it difficult to give the correct results through disc diffusion, E-test strips methods and agar dilution method.^12^ Clinical and Laboratory Standards Institute (CLSI) and European Committee on Antimicrobial Susceptibility Testing (EUCAST) has recommended the minimum inhibitory concentration (MIC) by micro broth dilution method for phenotypic identification of colistin resistance.^13^,^14^

Increasing resistance of gram negative bacteria and emergence of plasmid-mediated colistin resistance is the threat for this last-resort antibiotic which was also identified in Karachi and Peshawar, Pakistan.^4^,^15^ Therefore, this study was designed to identify the colistin resistance in uropthaogenic gram negative bacteria from Hayatabad Medical Complex, Peshawar.

## METHODS

This cross-sectional descriptive study was carried out in Hayatabad Medical Complex (HMC), Peshawar from January to June 2019. Ethical clearance was taken from the institutional ethical review board (IREB) of HMC Peshawar with reference number.144/HEC/B&PSC/19 dated 16 January 2019.

Intrinsically resistant bacteria such as *Proteus* spp., *Providencia* spp., *Morganella morganii*, *Pseudomonas mallei*, *Serratia marcescens*, *Chromobacterium* spp., *Burkholderia cepacia*, *Edwardsiella* spp., *Campylobacter*, *Brucella*, *Legionella*, and *Vibrio cholera* were excluded from study.

The patients were fully informed of the nature and purpose of the study before taking written informed consent. Urine sample was collected after taking informed consent from the patient/ attendant. Mid-stream urine samples were collected from infected patients in sterile urine collection bottles. Urine samples were transported to the Microbiology Laboratory of Health Research Institute, NIH, Research Centre, Khyber Medical College, Peshawar. Urine samples were streaked on Nutrient agar (Oxoid Limited, UK), MacConkey agar (Oxoid Limited, UK), SS agar (Oxoid Limited, UK), EMB agar (Oxoid Limited, UK), CLED (Sigma-Aldrich, Germany), CLED with android indicator (Oxoid Limited, UK) media and incubated under aerobic condition at 37c° for 24hrs. Gram negative bacteria were identified through Gram staining and further confirmation was done through API 10s system (bioMérieux, France). Modified Kirby Bauer disc diffusion method was used for antibiotic sensitivity and resistance testing. The bacterial growth was adjusted in sterile saline water to 0.5 McFarland standard solutions and streaked on Muller Hinton agar (Oxoid Limited, UK) for antibiogram. Results were interpreted according to Clinical and Laboratory Standards Institute (CLSI) guidelines.^14^

Minimal inhibitory concentration (MIC) was performed by broth microdilution method^17^ using 96-well round bottom microtiter plates (nest ®, Wuxi NEST Biotechnology Co., Ltd). Bacterial suspension was made using 4-6 colonies of bacterial growth in cation adjusted Muller Hinton Broth (Sigma-Aldrich, Germany). Bacterial suspension was adjusted to 0.5 McFarland standard which have bacterial concentration of 1 to 2 x10^8^ CFUml^-1^. Bacterial suspension was diluted in 1:20 ratio to achieve the final concentration of bacterial growth to 1×10^6^ CFU ml^-1^.^14^

Stock solution of Colistin sulphate (Sigma Aldrich St. Louis, MO, USA) was prepared according to CLSI, in sterile distilled water. Further serial two-fold dilutions of colistin were made in microtiter plate wells containing 50 *μ*L sterile CAMHB medium ranging 0.25 μg ml^-1^ to 64 μg ml^-1^. Prepared 50 *μ*L bacterial suspension was added to each well except negative control.

No growth was recorded as lowest concentration of MIC. MBC was performed by sub-culturing the 10 *μ*L of each well from MIC of microtiter plate on MHA (Oxoid Limited, UK) and incubated for 24hrs at 37 C° under aerobic condition. MBC was considered as lowest concentration that no growth was found on MHA media. The MIC range for colistin resistance of Enterobacteriaceae and *Pseudomonas* spp. was set as ≥4 μg ml^-1^ according to CLSI breakpoints.^14^

### Quality Control:

To assure the colistin resistance through MIC, reference strains (mcr1-mcr5) were used as a positive control which were provided by the Rene S Hendriksen (Technical University of Denmark, Anker Engelunds Vej 1DK-2800 Kgs. Lyngby, Denmark CVR. No. 30 06 09 46).

### Statistical Analysis:

All the data was entered and analyzed using SPSS version.20. Qualitative data was analyzed as frequency and percentage.

## RESULTS

**Fig.1:**
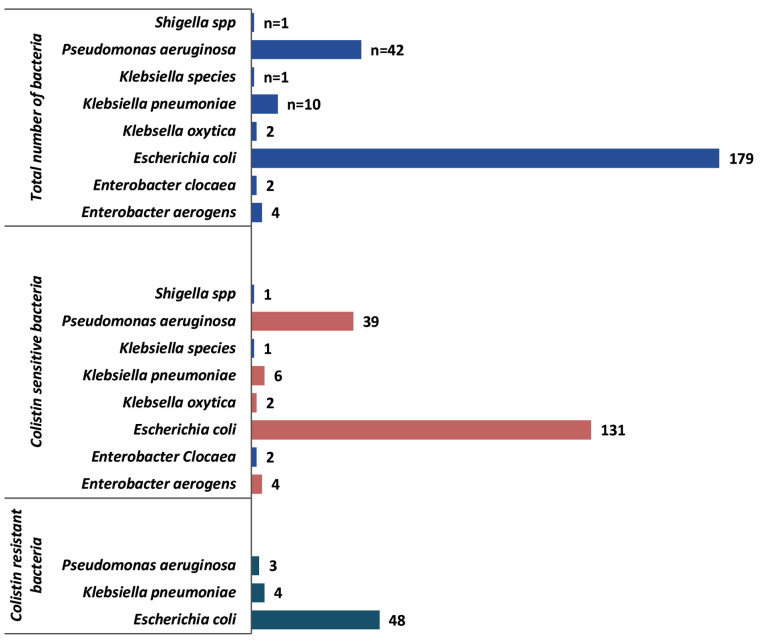
Frequency of uropathogenic gram negative bacterial isoltes (n=241).

A total of 2000 urine samples were collected, among which 281(14%) samples showed significant growth of gram-negative bacteria. Forty isolates were intrinsically resistant to colistin therefore they were excluded from this study. Among 241 positive samples, 134(55.6%) female and 107(44.4%) male were infected with uropthogenic bacteria. The most prevalent uropathogenic gram negative bacteria were *E.coli* 179(74.3%), followed by *Pseudomonas aeruginosa* 42 (17.5%) as shown in Fig.1. A total of 55(22.8%) colistin resistant bacterial isolates were identified through broth micro-dilution, where *E.coli* (n=48) was predominant followed by *Klebsiella pneumoniae* (n=4) and *Pseudomonas aeruginosa* (n=3).

Antibiogram of colistin resistant bacteria revealed that *E.coli* (n=48) showed resistance to Levofloxacin (81.2%), Ciprofloxacin (81.2%), Cefotaxim (77.1%), Ceftrioxone (77.1%), Amoxicillin/clavulanic acid (70.8%), Ceftazidim (66.7%) and Azteronam (62.5%). Colistin resistant *E.coli* showed emerging resistance to the Imepienum 16.7% and Meropenum 14.6% (Table-I).

**Table-I:** Antibiotic sensitivity and resistant profile of colistin resistant bacteria (n=55).

Antibiotics	Escherichia coli(n=48)		Klebsiella pneumoniae(n=4)		Pseudomonas aeruginosa(n=3)	

	R	S	R	S	R	S
Amikacin	10(20.9%)	38(79.1%)	2(50%)	2(50%)	0	3(100.0%)
Gentamycin	24(50%)	24(50%)	2(50%)	2(50%)	1(33.3%)	2(66.7%)
Imepienum	8(16.7%)	40(83.3%)	1(25%)	3(75%)	0	3(100.0%)
Meropenum	7(14.6%)	41(85.4%)	3(75%)	1(25%)	0	3(100.0%)
Cefepime	24(50%)	24(50%)	3(75%)	1(20%)	1(33.3%)	2(66.7%)
Ceftazidim	32(66.7%)	16(33.3%)	4(100%)	0	1(33.3%)	2(66.7%)
Ceftrioxne	37(77.1%)	11(22.9%)	4(100%)	0	1(33.3%)	2(66.7%)
Cefotaxim	37(77.1%)	11(22.9%)	4(100%)	0	1(33.3%	2(66.7%)
Azteronam	30(62.5%)	18(37.5%)	3(75%)	1(20%)	1(33.3%)	2(66.7%)
Amoxicillin and clavulanic acid	34(70.8%)	14(29.2%)	4(100%)	0	1(33.3%)	2(66.7%)
Piperacillin/tazo	11(22.9%)	37(77.1%)	1(25%)	3(75%)	0	3(100.0%)
Ciprofloxacin	39(81.2%)	9(18.8%)	4(100%)	0	0	3(100.0%)
Levofloxacin	39(81.2%)	9(18.8%)	4(100%)	0	1(33.3%)	2(66.7%)

Colistin resistant *Klebsiella pneumoniae* (n=4) showed complete (100%) resistance to Ceftazidim, Cefotaxim, Ceftrioxone, Amoxicillin/clavulanic acid, Levofloxacin and ciprofloxacin. Moreover, colistin resistant *Klebsiella pneumoniae* were resistant to Imepienum (25%), Meropenum (75%), Azteronam(75%) and Piperacillin/tazobactum (25%).

Colistin resistant *Pseudomonas aeruginosa(n=3)* was completely (100%) sensitive to Amikacin, Imipenum, Meropenum, Pipercillin/Tazobactum and Ciprofloxacin. Colistin MIC was performed by broth micro-dilution for isolated bacterial samples. A total of 48 colistin resistant *E.coli* had MICs range of 4 μg/ml to 64 μg/ml. *Klebsiella pneumonia*e (n=4) showed MIC range of 4 μg/ml to 64 μg/ml. *Pseudomonas aeruginosa* (n=3) have MICs from 8 μg/ml to 64 μg/ml.

Minimum bacterial activity was observed in *Escherichia coli* (n=41), *Enterobacter aerogens* (n=1), *Klebsiella pneumoniae* (n=2), *Klebsiella species* (n=1) and *Pseudomonas aeruginosa* (n=10). Majority of colistin sensitive uropathogenic gram negative bacteria were killed below 4 μg/ml of colistin concentration.

## DISCUSSION

Urinary tract infections(UTI) are mostly caused by bacteria, affecting 150 million people each year worldwide^16^ which needs more medical attention.^17^ In our study it was found that prevalence of UTI was 14% associated with Gram Negative Bacteria. Our study results were similar with studies done in Kohat which showed the 11.6% prevalence^18^ and 12.06% of UTI prevalence in Karachi.^17^ According to present study female (55.6%) were more affected with UTI which is comparable to other study done in Pakistan.^19^ While other study done in Pakistan results were higher (87.94%) than this study.^17^

**Table-II:** Colistin MIC^BMD^ (μg/ml) distributions among isolated uropathogenic Gram Negative Bacteria.

Colistin MIC_BMD_(μg/ml) range											

Bacterial isolates	No. of isolate	0	.25	.50	1	2	4	8	16	32	64
Enterobacter aerogens	4	1	1	0	2	0	0	0	0	0	0
Enterobacter clocaea	2	0	0	0	0	2	0	0	0	0	0
Escherichia coli	179	41	10	32	47	1	12	5	14	4	13
Klebsella oxytica	2	0	0	0	1	1	0	0	0	0	0
Klebsiella pneumoniae	10	2	1	1	1	1	0	0	3	0	1
Klebsiella species	1	1	0	0	0	0	0	0	0	0	0
Pseudomonas aeruginosa	42	10	1	11	14	3	0	1	0	1	1
Shigella spp	1	0	0	0	0	1	0	0	0	0	0

**Table-III:** Minimum bactericidal activity (μg/ml) of Colistin distributions among isolated uropathogenic gram negative bacteria.

Colistin MBC_BMD_(μg/ml) range											

Bacterial isolates	No. of isolate	0	0.5	1	2	4	8	16	32	64	128
Enterobacter aerogens	4	1	1	1	0	1	0	0	0	0	0
Enterobacter clocaea	2	0	0	0	0	1	1	0	0	0	0
Escherichia coli	179	41	27	23	35	12	2	15	9	13	2
Klebsella oxytica	2	0	0	1	1	0	0	0	0	0	0
Klebsiella pneumoniae	10	2	2	0	2	0	0	1	2	0	1
Klebsiella species	1	1	0	0	0	0	0	0	0	0	0
Pseudomonas aeruginosa	42	10	6	12	10	1	0	1	0	2	0
Shigella spp	1	0	0	0	1	0	0	0	0	0	0

*E.coli* (74.3%) was the leading cause of UTI in this study. Similar kind of results was also observed in other studies^17^-^19^ in which *E.coli* was major cause of UTI. Results of this study revealed 22.8% prevalence of colistin resistance. A study done in Pakistan showed 15.9% resistance to colistin in gram negative bacteria.^15^ Another study in Islamabad also showed similar results.^20^

In our study out of 55 colistin resistant bacteria, 87.2% *E.coli* and 7.3% *Klebsiella pneumoniae* were found to be resistant to colistin while a study done in Karachi showed that 7.5% *E.coli and* 50% *Klebsiella pneumonia*e were resistant to colistin.^15^ Another study done in Peshawar indicated that 40% *Klebsiella pneumonia*e and 23% *E.coli* showed resistance to colistin.^4^

Colistin resistant *E.coli* showed resistance to ciprofloxacin 81.2%, Amoxicillin and clavulanic acid 70.8%, Ceftazidim 66.7%, Cefepime 50% and Cefotaxim 77.1% in this study. These results are comparable to other study done in china.^21^

Colistin resistant *Klebsiella pneumoniae* isolates were 100% resistant to Ceftazidim, Cefotaxim, Ceftrioxone, Amoxicillin/clavulanic acid, Levofloxacin and ciprofloxacin. While resistance to Meropenum (75%), Azteronam (75%) and Piperacillin/tazobactum (25%) was also found in Colistin resistant *Klebsiella pneumoniae*. Similar results of high resistance were also observed in other studies.^22^,^23^ A research done in Peshawar revealed that resistance of colistin resistant *Klebsiella pneumoniae* to other antibiotics were much less than our study.^4^ Higher resistance to Fluoroquinolones, Cephalosporins, Monobactams and Penicillin combinations in our society could be due to irrational use of these antibiotics for the treatment of UTIs.

Isolated colistin resistant *Pseudomonas aeruginosa* during this research showed susceptibility to all used antibiotics while other study showed higher resistance in *Pseudomonas aeruginosa* to cephalosporins and carbapenems.^24^

Colistin sensitive uropathogenic gram negative bacteria were resistant to Ceftazidim, Cefotaxim, Ceftrioxone, Amoxicillin/Clavulanic acid, Levofloxacin and Ciprofloxacin according this study. Our results were in agreement to other study done in Pakistan.^18^

Uropathogenic Gram negative Bacteria that have 4ug/ml of MIC, most of them were not able to grow on MHA media plate at 4 ug/ml of MIC. Our results also corroborate with other study.^25^ The lowest level of colistin was 4ug/ml which kill/inhibit the majority of bacterial isolates of this study.

### Limitation:

This study was done only in one hospital of Peshawar and only targeted the UTI patients, which is limitation of this study. It might increase our knowledge about the causative bacterial agent pattern of UTI, amitotic resistant profile and most important colistin resistance if samples are selected from different hospitals and from other infections.

## CONCLUSION

This study concluded that *E.coli* was the main causative agent of UTI. Colistin resistant *E.coli* and *Klebsiella pneumonia*e isolates were resistant to important antibiotics e.g. fluoroquinolones, cephalosporins, monobactams, carbapenems and penicillin combinations.

### Authors’ Contribution:

**AA:** Samples collection, analysis & manuscript writing, literature search, methodology, discussion. **IU:** Conceived the project, project design, manuscript writing and data analysis. Correspondence with the journal and takes the responsibility and is accountable for all aspects of the work in ensuring that questions related to the accuracy or integrity of any part of the work are appropriately investigated and resolved. **OU:** Statistical analysis and manuscript editing. **RZ:** Data collection and analysis, microbiological procedure.

## References

[1] Loho T, Dharmayanti A (2015). Colistin:An antibiotic and its role in multiresistant Gram-negative infections. Acta Med Indones.

[2] Farajnia S, Alikhani MY, Ghotaslou R, Naghili B, Nakhlband A (2009). Causative agents and antimicrobial susceptibilities of urinary tract infections in the northwest of Iran. Int J Infect Dis.

[3] Pirkani GS, Awan MA, Abbas F, Din M (2020). Culture and PCR based detection of bacteria causing urinary tract infection in urine specimen. Pak J Med Sci.

[4] Hameed F, Khan MA, Bilal H, Muhammad H, Rehman TU (2021). Detection of MCR-1 gene in multiple drug resistant escherichia coli and klebsiella pneumoniae in human clinical samples from Peshawar, Pakistan. Comb Chem High Throughput Screen.

[5] Li J, Nation RL, Turnidge JD, Milne RW, Coulthard K, Rayner CR (2006). Colistin:the re-emerging antibiotic for multidrug-resistant Gram-negative bacterial infections. Lancet Infect Dis.

[6] Bialvaei AZ, Samadi Kafil H (2015). Colistin, mechanisms and prevalence of resistance. Curr Med Res Opin.

[7] Knopp M, Babina AM, Gudmundsdóttir JS, Douglass MV, Trent MS, Andersson DI (2021). A novel type of colistin resistance genes selected from random sequence space. PLoS Genet.

[8] Tan T, Ng S (2006). The in-vitro activity of colistin in gram-negative bacteria. Singapore Med J.

[9] Park YK, Choi JY, Shin D, Ko KS (2011). Correlation between overexpression and amino acid substitution of the PmrAB locus and colistin resistance in Acinetobacter baumannii. Int J Antimicrob Agents.

[10] Liu Y-Y, Wang Y, Walsh TR, Yi L-X, Zhang R, Spencer J (2016). Emergence of plasmid-mediated colistin resistance mechanism MCR-1 in animals and human beings in China:A microbiological and molecular biological study. Lancet Infect Dis.

[11] Rebelo AR, Bortolaia V, Kjeldgaard JS, Pedersen SK, Leekitcharoenphon P, Hansen IM (2018). Multiplex PCR for detection of plasmid-mediated colistin resistance determinants, mcr-1, mcr-2, mcr-3, mcr-4 and mcr-5 for surveillance purposes. Eurosurveillance.

[12] Poirel L, Jayol A, Nordmann P (2017). Polymyxins:antibacterial activity, susceptibility testing, and resistance mechanisms encoded by plasmids or chromosomes. Clin Microbiol Rev.

[13] Testing ECoAS (2016). Recommendations for MIC determination of colistin (polymyxin E) as recommended by the joint CLSI-EUCAST Polymyxin Breakpoints Working Group. EUCAST:Vaxjo, Sweden.

[14] CLSI (2018). Methods for Dilution Antimicrobial Susceptibility Tests for Bacteria That Grow Aerobically, 11th Edition. CLSI Standard M07.

[15] Qamar S, Shaheen N, Shakoor S, Farooqi J, Jabeen K, Hasan R (2017). Frequency of colistin and fosfomycin resistance in carbapenem-resistant Enterobacteriaceae from a tertiary care hospital in Karachi. Infect Drug Resist.

[16] Medina M, Castillo-Pino E (2019). An introduction to the epidemiology and burden of urinary tract infections. Ther Adv Urol.

[17] Zubair KU, Shah AH, Fawwad A, Sabir R, Butt A (2019). Frequency of urinary tract infection and antibiotic sensitivity of uropathogens in patients with diabetes. Pak J Med Sci.

[18] Ullah A, Shah S, Almugadam B, Sadiqui S (2018). Prevalence of symptomatic urinary tract infections and antimicrobial susceptibility patterns of isolated uropathogens in kohat region of Pakistan. MOJ Biol Med.

[19] Ali G, Riaz-Ul-Hassan S, Sadia MAS, Javid MQ, Khan AR, Shakir L (2020). Antibiotic susceptibility and drug prescription pattern in uropathogenic Escherichia coli in district Muzaffarabad, Azad Jammu and Kashmir, Pakistan. J Pak Med Assoc.

[20] Imtiaz W, Syed Z, Rafaque Z, Andrews SC, Dasti JI (2021). Analysis of Antibiotic Resistance and Virulence Traits (Genetic and Phenotypic) in Klebsiella pneumoniae Clinical Isolates from Pakistan:Identification of Significant Levels of Carbapenem and Colistin Resistance. Infect Drug Resist.

[21] Wang Y, Tian G-B, Zhang R, Shen Y, Tyrrell JM, Huang X (2017). Prevalence, risk factors, outcomes, and molecular epidemiology of mcr-1-positive Enterobacteriaceae in patients and healthy adults from China:An epidemiological and clinical study. Lancet Infect Dis.

[22] Pena I, Picazo JJ, Rodriguez-Avial C, Rodriguez-Avial I (2014). Carbapenemase-producing Enterobacteriaceae in a tertiary hospital in Madrid, Spain:high percentage of colistin resistance among VIM-1-producing Klebsiella pneumoniae ST11 isolates. Int J Antimicrob Agents.

[23] Kontopoulou K, Protonotariou E, Vasilakos K, Kriti M, Koteli A, Antoniadou E (2010). Hospital outbreak caused by Klebsiella pneumoniae producing KPC-2 β-lactamase resistant to colistin. J Hosp Infect.

[24] Wi YM, Choi JY, Lee JY, Kang CI, Chung DR, Peck KR (2017). Emergence of colistin resistance in Pseudomonas aeruginosa ST235 clone in South Korea. Int J Antimicrob Agents.

[25] Jayol A, Nordmann P, Brink A, Poirel L (2015). Heteroresistance to colistin in Klebsiella pneumoniae associated with alterations in the PhoPQ regulatory system. Antimicrob Agents Chemother.

